# Virus Detection: From State‐of‐the‐Art Laboratories to Smartphone‐Based Point‐of‐Care Testing

**DOI:** 10.1002/advs.202105904

**Published:** 2022-04-07

**Authors:** Meng Xiao, Feng Tian, Xin Liu, Qiaoqiao Zhou, Jiangfei Pan, Zhaofan Luo, Mo Yang, Changqing Yi

**Affiliations:** ^1^ Guangdong Provincial Key Laboratory of Sensing Technology and Biomedical Instrument, School of Biomedical Engineering Shenzhen Campus of Sun Yat‐Sen University Shenzhen 518107 P. R. China; ^2^ Department of Biomedical Engineering The Hong Kong Polytechnic University Hunghom Hong Kong 999077 P. R. China; ^3^ Department of Clinical Laboratory The Seventh Affiliated Hospital of Sun Yat‐Sen University Shenzhen 518107 P. R. China

**Keywords:** biosensors, laboratory‐based diagnostics, point‐of‐care testing, smartphones, virus

## Abstract

Infectious virus outbreaks pose a significant challenge to public healthcare systems. Early and accurate virus diagnosis is critical to prevent the spread of the virus, especially when no specific vaccine or effective medicine is available. In clinics, the most commonly used viral detection methods are molecular techniques that involve the measurement of nucleic acids or proteins biomarkers. However, most clinic‐based methods require complex infrastructure and expensive equipment, which are not suitable for low‐resource settings. Over the past years, smartphone‐based point‐of‐care testing (POCT) has rapidly emerged as a potential alternative to laboratory‐based clinical diagnosis. This review summarizes the latest development of virus detection. First, laboratory‐based and POCT‐based viral diagnostic techniques are compared, both of which rely on immunosensing and nucleic acid detection. Then, various smartphone‐based POCT diagnostic techniques, including optical biosensors, electrochemical biosensors, and other types of biosensors are discussed. Moreover, this review covers the development of smartphone‐based POCT diagnostics for various viruses including COVID‐19, Ebola, influenza, Zika, HIV, et al. Finally, the prospects and challenges of smartphone‐based POCT diagnostics are discussed. It is believed that this review will aid researchers better understand the current challenges and prospects for achieving the ultimate goal of containing disease‐causing viruses worldwide.

## Graphical abstract

This review summarizes the latest development of virus detection techniques, with the special focus on smartphone‐based viral diagnostics whose advances have enabled laboratory‐based molecular detections to be performed with plug‐and‐play stand‐alone devices. Discussion on challenges and future perspectives of smartphone‐based viral diagnostics is highlighted, which might push the development of mobile diagnostics forward with scientific and available guidance.

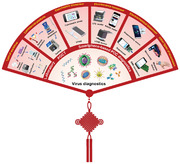

## 1 Introduction

Emerging viruses present one of the greatest public health threats facing human populations. The outbreak of Coronavirus Disease 2019 (COVID‐19) has evolved into a global crisis, which has been defined as a public health emergency of international concern by the World Health Organization (WHO). As of February 25, 2022, authorities in 206 countries and territories had reported over 431 million cases, resulting in at least 5.9 million deaths worldwide. Controlling the spread of these viruses continues to be a global challenge. China has been the most successful country in containing the epidemic and its successful experience lies in early detection for early control. Early detection of virus infection is critical for virus containment because it can effectively identify suspected individuals and cut off the transmission chains. In addition, early detection of virus infection is critical in tracing transmission chains, such as "who‐infected‐whom," contact tracing, and human‐to‐human transmission timelines, thereby assisting in interrupting the spread of virus.

Viruses are composed of nucleic acids (DNA or RNA) and an envelope protein coat. They typically require a host cell to replicate their genomes and multiply virus particles. Based on this unique characteristic, molecular diagnostic methods such as virus isolation,^[^ ^1^, ^2^ ^]^ enzyme‐linked immunosorbent assay (ELISA),^[^ ^3^, ^4^ ^]^ polymerase chain reaction (PCR)^[^ ^4^, ^5^ ^]^ and hemagglutination/inhibition^[^ ^5^ ^]^ assay have been developed for virus identification and/or quantitation. These traditional techniques are generally performed in the central laboratory and hospital as the preferred diagnostic methods. However, these laboratory‐based tests necessitate not only sophisticated equipment but also highly trained operators, which are not easily deployed in the field. The recent COVID‐19 epidemic demonstrates that laboratory‐based testing alone is insufficient to prevent epidemic outbreaks, even in developed countries. Furthermore, laboratory‐based testing is nearly impossible for the majority of people in regions with scarce resources. As a result, limited testing capacity slowed the response to the COVID‐19 epidemic, eventually resulting in a global public health crisis.

Point‐of‐care testing (POCT), an emerging diagnostic platform, provides a clinically relevant testing method that has significantly improved on‐site infectious disease management and monitoring in recent years.^[^ ^6^ ^]^ WHO coined the term “ASSURED” to refer to an ideal POCT device that is affordable, sensitive, specific, user‐friendly, rapid, equipment‐free, and delivered. Additionally, as an alternative for laboratory‐based testing, the unique advantages of POCT for virus disease outbreak control include: 1) simpler fabrication and operation; 2) less analysis time; and 3) reduced reagent consumption. It should be emphasized that early detection of viral diseases can reduce disease infectiousness and mortality. A survey indicated that point‐of‐care (POC) malaria diagnostic could save more than 22% of lives compared to diagnosis based on laboratory infrastructure.^[^ ^7^ ^]^ As a result, WHO encourages the development and investment in research of POCT for infectious disease diagnostics,^[^ ^8^ ^]^ which will benefit not only district hospitals and centralized labs, but also rural clinics with inadequate infrastructure.

Currently, various commercial POCT devices have been developed for the purpose of detecting early pandemic outbreaks. Innovative advances in microfluidics, microelectron‐mechanical systems (MEMS) technology, nanotechnology, and 3D printing, as well as data analytics, have facilitated the development of POCT diagnosis significantly.^[^ ^9^, ^10^ ^]^ Based on these technologies, POCT devices have been widely reported and commercialized.^[^ ^11^, ^12^ ^]^ These devices include lab‐on‐a‐chip/disc (LOC/LOAD) devices, lateral flow devices, microfluidic paper‐based analytical devices (*μ*PADs) and isothermal nucleic acid amplification devices (INAA). And more notably, technological advances in mobile communications, low‐cost optical devices and accessible electronics have increased the speed and efficiency of data collection, processing and transmission globally.^[^ ^13^ ^]^

As a technological marvel, smartphones feature advanced high‐resolution digital cameras, computing, and storage capabilities, as well as advanced processors and an open‐source operating system, all of which enable them to incorporate promising technologies for detection, processing, and transmitting/receiving test results for POC testing.^[^ ^6^, ^14^ ^]^ Additionally, the global smartphone penetration rate as a percentage of the total population was 49.35% in 2016 and was projected to reach 78.05% by 2020.^[^ ^9^ ^]^ Thus, the smartphone is a ubiquitous device that is well‐suited for POC diagnostics and personalized healthcare, which may aid in the control of epidemic diseases.^[^ ^15^, ^16^ ^]^ Also, the excellent functional extendibility of smartphones through the use of peripheral attaching accessories or accessory‐free systems enables smartphones to accurately measure a variety of signals, including colorimetric,^[^ ^17^ ^]^ fluorescent,^[^ ^18^ ^]^ chemiluminescent,^[^ ^19^ ^]^ surface‐enhanced Raman scattering (SERS),^[^ ^20^ ^]^ and surface plasmon resonance (SPR) signals.^[^ ^21^ ^]^ Moreover, smartphones can be combined with microscopic and electrochemical methods to achieve a clinically acceptable level of diagnosis.^[^ ^22^, ^23^ ^]^ Without a doubt, the development of mobile POC tests expands the potential applications for diagnosing and monitoring infectious pathogens, as well as improves personalized healthcare in clinical management.

In this review, we focus on recent efforts to adopt smartphone technology for viral disease diagnostics, monitoring, and management. First, advances in POCT are compared to those in conventional laboratory‐based molecular diagnostics. Then, we highlight the current various smartphone‐based diagnostic technologies based on different sensing mechanisms, including immune sensing and nucleic acid testing. Moreover, this review covers the advances in POC diagnostics technology for COVID‐19, Ebola, Dengue, Zika, Human Immunodeficiency Virus (HIV), Hepatitis, and Influenza using smartphones. Additionally, future developments of smartphone‐based POC diagnostics is discussed, including associated perspectives and challenges. This review will aid researchers in comprehending the rapid development of smartphone‐based POC diagnostics toward the ultimate goal of curbing disease‐causing viruses worldwide.

## 2 Virus Diagnostics from Centralized Laboratories to Point‐of‐Care Testing

### 2.1 The Spread and Infection of Viruses

**Figure 1:**
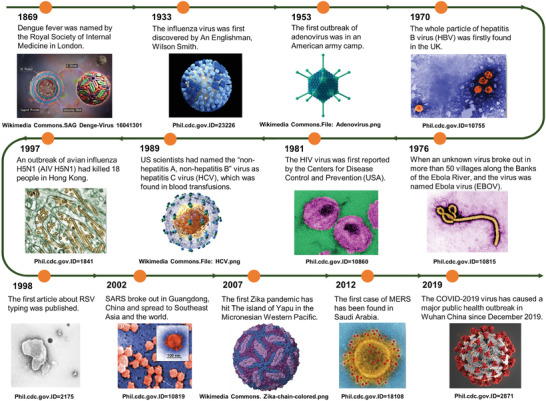
The appeared of major events in popular infectious diseases. From top to bottom are digitally colorized transmission electron microscopic (TEM) or 3D particle structure images of various infectious viruses. Image of Adonavirus: https://commons.wikimedia.org/wiki/File:Adenovirus_3D_schematic.png. Image of HCV: https://commons.wikimedia.org/wiki/File:HCV.png. Image of Zika Virus: https://commons.wikimedia.org/wiki/File:Zika-chain-colored.png.

Zoonotic viruses become prevalent in human communities periodically, resulting in disease syndromes of varying severity.^[^ ^24^, ^25^ ^]^ Throughout human history, viruses have placed a significant burden on public healthcare and resulted in significant economic and humanitarian losses (**Figure** **1**).^[^ ^26^ ^]^ A newly discovered virus can cause a localized outbreak or, in the worst‐case scenario, a large epidemic or global pandemic, depending on its infectivity in humans.^[^ ^27^, ^28^ ^]^ On a global scale, these emergence events have resulted in significant morbidity, mortality, and economic costs for humans.^[^ ^29^ ^]^ Several pandemics have occurred over the last two centuries, including the first report of dengue viruses in 1869, the severe acute respiratory syndrome (SARS) epidemic in Asia in 2003,^[^ ^30^ ^]^ the H1N1 influenza outbreak in 2009 in North America,^[^ ^31^ ^]^ and the Ebola virus epidemic in West Africa in 2014,^[^ ^32^ ^]^ as well as the ongoing global Zika virus,^[^ ^33^ ^]^ dengue virus,^[^ ^34^ ^]^ HIV,^[^ ^35^ ^]^ hepatitis B virus (HBV),^[^ ^36^ ^]^ swine‐ and avian‐origin influenza (AIV) crisis.^[^ ^37^ ^]^

**Figure 2:**
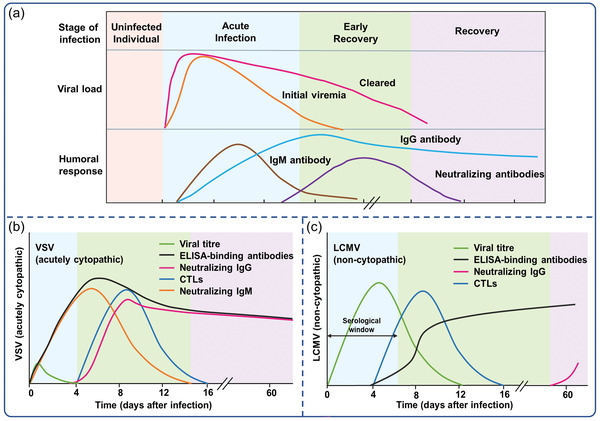
a) Immune response to virus infection, time points are approximate and could be changed depending on the different viruses. Protective immune responses of adaptive immune system to b) acutely cytopathic viruses and c) poorly cytopathic viruses. Reproduced with permission.^[^ ^42^ ^]^ Copyright 2006, Nature Publishing Group.

If the progression and pathogenesis of viral infections are tracked, it is discovered that once the virus enters the human body, it usually enters cells via interactions with cell surface receptors such as HIV‐related CD4 molecules,^[^ ^38^ ^]^ AIV‐related hemagglutinin (HA),^[^ ^39^ ^]^ and COVID‐19‐related angiotensin‐converting enzyme 2 (ACE2) receptor.^[^ ^40^ ^]^ Early in infection, viruses replicate themselves using host cell biological substances, and infect new cells, but which is first monitored by CD8+ T cell‐mediated immunity mechanisms.^[^ ^41^ ^]^ CD8+ T cells (cytotoxic T cells) are capable of killing infected cells at this stage, resulting in viral release from host cells but a lower viral load in the blood or tissue (**Figure** **2**a). After several days of infection, humoral immune responses, such as serum IgM antibody and subsequent IgG antibody, begin to appear.^[^ ^41^ ^]^ The entire stage of infection is associated with the virus's infectiousness. It is worth noting that patients with mild transient symptoms are frequently overlooked until the onset of severe or unusually symptomatic patients, but the infection may have spread long enough to cause a serious population infection in the meantime. Thus, this underestimation of asymptomatic individuals highlights the critical nature of virus diagnostics, particularly early screening.

### 2.2 Virus Diagnosis Principles and Associated Centralized Laboratories

Despite enormous efforts and significant progress, reducing infectious disease spread and mortality remains a global challenge, particularly in resource‐limited settings.^[^ ^34^ ^]^ Identifying the pathogens that cause disease and detecting infected patients is critical for disease prevention and treatment monitoring. Typically, virus infection mainly includes acutely cytopathic viruses such as rabies virus and smallpox virus,^[^ ^42^ ^]^ and poorly cytopathic viruses such as HBV,^[^ ^43^ ^]^ HCV,^[^ ^44^ ^]^ and HIV.^[^ ^35^ ^]^ In the case of acutely cytopathic infections, the ELISA‐binding antibodies typically persist for ∼2 weeks after infection. As with viruses that are poorly cytopathic, antibody kinetics are detected ∼5–8 days after infection (Figure 2b).^[^ ^42^ ^]^ Notably, virus infection has serological windows during which neither infection‐associated antibody nor pathogen‐specific antigen can be detected prior to host seroconversion (Figure 2c). The induction of broadly specific antibodies against HIV takes several weeks to months at a detectable level by immunoassay, which is significantly longer than the 3–7 days required for cytopathic viruses.^[^ ^45^ ^]^ Benefiting from gene sequencing technology, nucleic acid testing is capable of detecting infection in the early stage due to its high sensitivity and specificity. Thus, nucleic acid amplification tests (NAATs) can detect DNA/RNA of viruses directly, which is critical for early infection detection prior to seroconversion. Hence, NAATs have an advantage in detecting poorly cytopathic viruses early.

**Figure 3:**
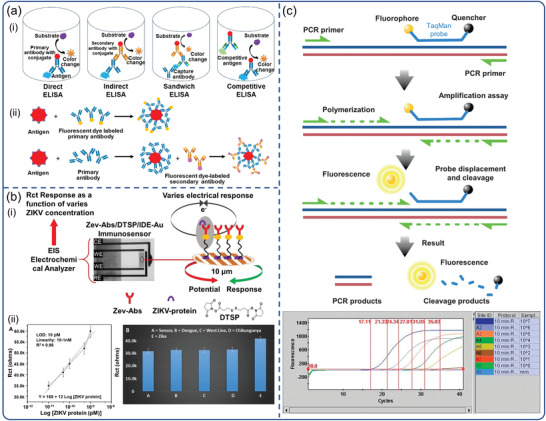
Laboratory‐based virus detection. Schematic diagram of the basic principle: a) ELISA. Reproduced with permission.^[^ ^46^ ^]^ Copyright 2016, Nexus Academic Publishers. b) electrochemical immunoassay. Reproduced with permission.^[^ ^47^ ^]^ Copyright 2018, Nature Publishing Group. c) RT‐PCR. Reproduced with permission.^[^ ^48^ ^]^ Copyright 2011, Elsevier.

Laboratory‐based diagnostic tools for virally infected individuals have been widely developed in response to the progression of the viral illness. Among them, the most commonly employed analytical techniques in clinics are those that analyze particular nucleic acids (RNA and DNA) and proteins (antibodies and antigens). Immunoassays are one of the most widely used serological methods for detecting viral antigens or serological antibodies following virus infection. They are cost‐effective and efficient due to the availability of high‐specific monoclonal antibodies and a variety of reporter modalities.^[^ ^46^, ^47^ ^]^ It is based on the detection of antigens by enzyme labeled antibodies, which enables the quantification of particular proteins (**Figure** **3**a,b). Immunoassays aid clinicians in tracking sick and recovered patients, assisting in the estimation of overall infections. This technique, however, has several drawbacks: 1) In comparison to the nucleic acid test, the immunoassay is not sensitive enough, making viral proteins difficult to detect. 2) Due to the window of time, serological antigens and antibodies may not be identified in the early stages. 3) Antibodies against viruses may react cross‐reactively with antibodies against other related viruses.

Because of its superior sensitivity and specificity, real‐time PCR has remained the gold standard for virus diagnosis up to now (Figure 3c).^[^ ^48^ ^]^ This technique is used in the majority of commercial kits (**Table** **1**). PCR, on the other hand, is time‐consuming due to the need of thermal cycling. As a result, isothermal amplification techniques are currently being developed to enable rapid and low‐cost nucleic acid detection with minimal instrumentation. Numerous isothermal techniques for virus detection have been developed or commercialized, including nuclear acid sequence‐based amplification (NASBA),^[^ ^49^, ^50^ ^]^ loop‐mediated amplification (LAMP),^[^ ^51^, ^52^ ^]^ rolling circle amplification (RCA),^[^ ^53^ ^]^ and strand displacement amplification (SDA).^[^ ^54^ ^]^ Related examples that have been commercialized are listed in Table 1. While a variety of commercial kits are available and could be used in clinics, all of these methods require a sophisticated infrastructure and centralized laboratories equipped with extremely expensive equipment and staffed by skilled personnel. Those immunoassay and NAATs analyzers capable of performing "sample‐in/answer‐out" testing are more automated, but unfortunately still highly complex and confined to central labs or hospitals. The demand for affordable, equipment‐free, and delivered testing methods is increasing, and as a result, new methods are being developed.

**Table 1:** Examples of commercial kits that are available to market for virus detection

Detection	Test kits	Target	Method	Amplification	Sensitivity^a)^	Time to result [min]^b)^	Manufacturer	Website
HIV‐1/HIV‐2 p24	RocheElecsys HIV Combi PT	p24 antigen	Sandwich ELISA	N/A	≤2 IU mL^−1^	27	Roche	www.roche.com
Dengue (serotype 1–4)	Abcam human anti‐dengue virus IgG ELISA	IgG	Indirect immunoassay	N/A	>90%	90	Abcam	www.abcam.cn
HBV	Bayer ADVIA Centaur HCV assay	HBsAg	Chemiluminescent immunoassay	N/A	0.12 ng mL^−1^	29	Bayer	www.bayer.com
Respiratory syncytial virus (RSV)	Anti‐Respiratory Syncytial Virus (RSV) IgM ELISA kit	RSV IgM	Enzyme immunoassay	N/A	N/A	90	Antibodies	www.antibodies‐online.cn
Avian influenza	ID Screen influenza competitive subtype‐specific kits	N/A	Competitive ELISA	N/A	93%	150	ID	www.id‐vet.com
ZIKV	TaqPath Zika Virus kit	N/A	Nucleic acid amplification	RT‐PCR	1 copies mL^−1^	<1 h	Thermofisher	www.thermofisher.com
MERS‐CoV	RealStar MERS‐CoV Kit	N‐gene	Nucleic acid amplification	RT‐PCR	2.8 × 10^−3^ PFU	<1 h	Altona	www.altona‐diagnostics.com
2019‐nCoV, SARS‐CoV	QIAGEN One‐Step RT‐PCR Kit (2019‐nCoV, SARS‐CoV)	E gene	Nucleic acid amplification	RT‐PCR	5.2 copies mL^−1^	<30 min	QIAGEN	www.Qiagen.com
		N gene			8.3 copies mL^−1^			
Influenza virus	ABI 7500 influenza A‐kit	M gene	Nucleic acid amplification	RT‐PCR	10–100 copies mL^−1^	<30 min	Thermofisher	www.thermofisher.com
RSV	BioRad virus RT‐PCR Kit	F gene of RSV	Nucleic acid amplification	RT‐PCR	1.4 × 10–1.4 × 10^6^ pg mL^−1^	3.5–4 h	Bio‐Rad	www.bio‐rad.com
HAdV HIV‐1 SARS‐CoV‐2 HSV1/HSV2	Altona RealStar Adenovirus PCR Kit 1.0 NucliSens HIV‐1 EasyQ 2.0 SARS‐CoV‐2 visual RT‐LAMP kit BD Viper LT	HAdV (hexon) N/A N gene N/A	Nucleic acid amplification Nucleic acid amplification Dye method Fluorescent energy transfer	One‐Step RT‐PCR NASBA LAMP SDA	<1.7 log_10_ copies mL^−1^ 10 copies mL^‐1^ 10 copies mL^−1^ 96.8%	2 h <3 h 40 min <4.5 h	Altona Biomeriex Biorab BD	www.altona‐diagnostics.com www.biomerieux.com.cn www.biorab.com www.bd.com

^a)^ International unit: IU (WHO International Standard Unit); plaque‐forming units: PFU; tissue culture infective dose: TCID;

^b)^ time to result depends upon the particular assay.

### 2.3 Point‐of‐Care Testing for Virus Diagnostics

**Figure 4:**
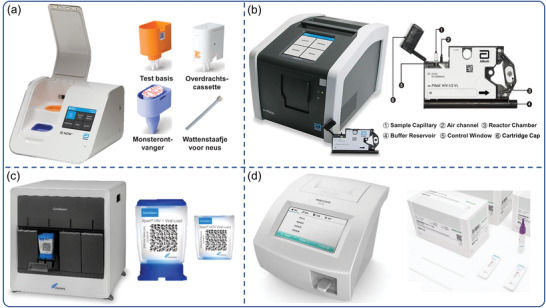
POC virus diagnostic systems and equipment currently marketed. a) ID NOW influenza A & B 2 system (Abbott Co., Ltd). Reproduced with permission.^[^ ^172^ ^]^ Copyright 2022, Abbott. b) The m‐PIMA analyzer and m‐the PIMA HIV‐1/2 test cartridge (Abbott Co., Ltd). Reproduced with permission.^[^ ^173^ ^]^ Copyright 2022, Abbott. c) The GeneXpert System and the Xpert HIV‐1/HCV viral load test cartridge (Cepheid Co., Ltd). Reproduced with permission.^[^ ^174^ ^]^ Copyright 2022, Cepheid. d) R01 fluorescence immunoassay analyzer and Rapid test strip (Maccura Co., Ltd). Reproduced with permission.^[^ ^175^ ^]^ Copyright 2022, Maccura.

As mentioned, many of the instruments and professional technology required for laboratory‐based virus diagnostics are unavailable during a large‐scale epidemic outbreak, particularly during a global pandemic. As a low‐cost, decentralized, and accessible platform, POCT enables the cost‐effective diagnosis of infected patients during epidemic outbreaks. Commercialization of POCT platforms continues to be a challenge due to the requirement for affordable, minimally trained personnel, and maintenance‐free instrumentation with robust, ready‐to‐use reagents. While POCT is still in its infancy on a global scale, technological advancements are beginning to address these issues. Some commercial POCTs have been developed to enable the simultaneous analysis of multiple samples, including quantitative polymerase chain reaction (q‐PCR) and high‐throughput immunoassays. These POCT platforms incorporated time‐saving, automated instruments and are primarily comprised of three steps: sample pretreatment, analyte capture/amplification, and detection. **Table** **2** lists commercially available examples of fully or partially integrated platforms. In the GeneXpert, m‐PIMA, Cobas MPX, Cobas Liat Influenza A/B, and other real‐time PCR‐based benchtop systems, integration of ready‐to‐use reagents and microfluidic chips with POCT virus diagnostic systems has been demonstrated (Table 2). Using isothermal LAMP, as implemented in the ID NOW influenza A & B 2 system (Abbott Co., Ltd), m‐PIMA analyzer (Abbott Co., Ltd) and GeneXpert System (Cepheid Co., Ltd), significantly reduces cost and complexity (**Figure** **4**a,c). Rapid test strips are facilitated by simple end‐point detection schemes in a minimal instrumentation manner, as demonstrated by R01 fluorescence immunoassay analyzer (Maccura Co., Ltd) (Figure 4d). We can see that commercial POCT platforms have evolved in two directions: 1) miniaturization of traditional laboratory‐based equipment, an emphasis on platform automation design, and a reduction in personnel requirements for operation; and 2) single‐use handheld devices benefit from advances in the field of micromanufacturing technology, which have combined complex modules such as sample processing, reagent storage and detection.

**Table 2:** Examples of commercial POCT platforms that are available to market for virus detection

Detection	Production	Target	Method	Amplification	Sensitivity^a)^	Time to result [min]^d)^	Manufacturer	Website
HIV‐1	m‐PIMA HIV‐1/2 VL	HIV‐1 group M, group N and group O, HIV‐2	Microfluidic chips	Real‐time qRT‐PCR	800 copies mL^−1^	<70 min	Abbott	www.abbott.com
HIV‐1	Xpert HIV‐1 VL	HIV‐1 group M, group N and group O	Microfluidic chips	Real‐time qRT‐PCR	40–10^7^ copies mL^−1^	<90 min	Cepheid	www.cepheid.com
HIV‐1/HIV‐2/HCV/HBV	Cobas TaqScreen MPX Test	N/A	Nucleic acid amplification	Multiple real‐time PCR	50 copies mL^−1^ (HIV),^b)^ 50 copies mL^−1^ (HCV), 10 copies mL^−1^ (HBV)^c)^	<60 min	Roche	www.roche.com
HIV‐1/HCV/HBV	Ultrio	N/A	Nucleic acid amplification	Multiple real‐time PCR	44.6 IU mL^−1^ (HIV), 10.44 IU mL^−1^ (HBV),3.01 IU mL^−1^ (HCV)	<60 min	Novartis	www.novartis.com
HIV‐1/HIV‐2	Alere q HIV‐1/2	HIV‐1 group M, group N and group O, HIV‐2	Nucleic acid amplification	Real‐time PCR	2937 IU mL^−1^	<60 min	Abbott	www.abbott.com
Influenza A/B	Cobas Liat Influenza A/B	N/A	Microfluidic chips	Real‐time PCR	10^−2^–10^−1^ TCID_50_ mL^−1^ (Influenza A)/10^−3^–10^−1^ TCID_50_ mL^−1^ (Influenza B)	15–20 min	Roche	www.diagnostics.roche.com
Influenza A/B	ID NOW INFLUENZA A & B 2	N/A	Microfluidic chips	Isothermal (RPA)	N/A	<13 min	Abbott	www.abbott.com
Influenza A/B	BD veritor system Flu A+B	N/A	Immunochromatographic strip	N/A	N/A	15–20 min	BD	www.bd.com
Ebola virus	Film array NCDS BT‐E	N/A	FilmArray NGDS BTE assay pouches	Real‐time qRT‐PCR	100 PFU mL^−1^	1 h	BioFire	www.biofireinc.com
Ebola virus	Lightcycler,COBAS Z480	N/A	High pure viral nuclenic acid Kit	Real‐time qRT‐PCR	4781 PFU mL^−1^	3 h	Roche	www.roche.com
Ebola virus	LA‐200	Nucleoprotein coding genes	Nucleic acid amplification	Isothermal (LAMP)	10^4^–10^5^ copies mL^−1^	<60 min	Eiken	www.eiken.co.jp
HIV	LIAISON 200 tests 310260 XL murex HIV Ab/Ag	p24 antigen	Chemiluminescence immunoassay	N/A	27.4 pg mL^−1^	<60 min	Diasorin	www.diasorin.com
HIV‐1/HIV‐2	HIV(I+2) rapid test strip	N/A	Rapid test strip	N/A	N/A	<15 min	Kehua Bio‐engineering	www.skhb.com
Syphilis	Rapid test strip	Syphilis antigen	Rapid test strip	N/A	N/A	15–20 min	Wondfo	www.wondfo.com.cn
Ebola virus	ReEBOV antigen rapid test	Antigen VP40	Rapid test strip	N/A	1.0 × 10^6^ PFU mL^−1^	<15 min	Corgenix	www.corgenix.com
Ebola virus	OraQuick Ebola rapid antigen test	Antigen	Rapid test strip	N/A	1.64 × 10^6^ PFU mL^−1^	30 min	OraSure	www.oraSure.com

^a)^ International unit: IU (WHO International Standard Unit); Plaque‐forming units: PFU; Tissue culture infective dose: TCID;

^b)^ diagnostic sensitivity at limit of detection with WHO 3rd International Standard, 1000 copies mL^−1^;

^c)^ 1 IU mL‐−1 ∼  5.6 copies mL^−1^, which is defined by the WHO;

^d)^ time to result depends upon the particular assay.

Recently, researchers have endeavored to address these POCT systems characteristics by utilizing advances in MEMS,^[^ ^55^ ^]^ microfluidics,^[^ ^56^, ^57^ ^]^ field‐effect transistor (FET),^[^ ^58^, ^59^ ^]^ and photoacoustic (PA) microscope technology.^[^ ^60^, ^61^ ^]^ Microfluidic chips provide a microscale biochemical reaction platform at the micrometer scale compatible with compact instrumentation or a simple handheld reader, minimizing reagent consumption and reducing test costs. Furthermore, LOC/LOAD devices integrated sample pretreatment, biochemical reaction, and detection on a microfluidic chip, shortens testing time and enables field‐deployment.^[^ ^62^ ^]^ For example, the hybrid optofluidic integration of microfluidic sample preparation multiplexer (SPM) and optical sensing significantly simplified processing, achieving in “sample‐to‐answer” formats without manual intervention.^[^ ^63^ ^]^ To improve sensitivity and enable multiplexed analysis, an increasing number of novel nanomaterials have facilitated the development of POCT, such as nanomaterials‐based electrochemical and optical biosensors. In particular, the integration of graphene‐based FET with clustered regularly interspaced short palindromic repeats (CRISPR) technology not only significantly enhances sensitivity in output signal, but also extends amplification‐free techniques.^[^ ^58^ ^]^ Moreover, PA microscopy is an emerging imaging technology that relies on laser‐generated photoacoustic effect by light‐absorbing molecules due to adiabatic expansion.^[^ ^64^ ^]^ In PA imaging, a variety of endogenous molecules (e.g., DNA, RNA, hemoglobin, and lipids) and exogenous contrast agents (e.g., nanoparticles) can be mapped with a high resolution and desired penetration depths.^[^ ^60^ ^]^ To date, technological advances in PA microscopy have undergone a revolution in portability and wearability, which may offer new paradigms for POC virus diagnostic in the future.

**Figure 5:**
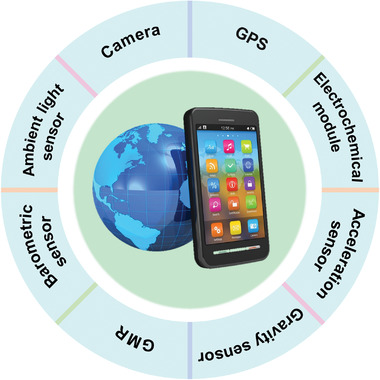
Built‐in functional modules in smartphones include the camera, ambient light sensor, distance sensor, gravity sensor, acceleration sensor, electrochemical module, magnetic sensor, barometric sensor, gyroscopes, global position system (GPS).

While those POCT systems appear to be exciting, a review of infectious disease diagnosis reveals that 75% of research continues to rely on laboratory resources (centrifuges, pipettes) to run molecular assays and trained users to interpret results.^[^ ^10^ ^]^ To minimize user error and maximize clinical impact in a resource‐limited setting, automated result analysis should be considered for interpreting, recording, and transmitting test results. As a result, the POCT system must be embedded with corresponding functional modules, which increases the difficulty of transporting the POCT system and complicating data collection and analysis. A smartphone is a small, portable, multi‐functional communication device that incorporates a variety of functional modules such as a camera, ambient light sensor, electrochemical module, magnetic sensor, barometric sensor, gravity sensor, acceleration sensor, global position system (GPS), and global mobile communication (GSM) (**Figure** **5**). Due to their widespread use and “smart” capabilities, smartphones enable advanced POCT systems, effectively facilitating and expediting clinical diagnosis of viral disease.^[^ ^9^ ^]^ These smartphone‐based devices either capitalize the phone's built‐in sensors, such as the camera, ambient light sensor, electrochemical module, magnetic sensor, and barometric sensor, or utilize external sensor modules connected via wired or wireless connections, to seamlessly integrate into a connected diagnostic system.^[^ ^24^ ^]^ Smartphones can be used as a controller, analyzer, and displayer for portable and mobile sensing systems by taking advantages of their built‐in function modules. For instance, the smartphone camera is able to capture color images of test samples and transmit the acquired image to the test results via custom apps. A smartphone camera can also replace a laboratory‐based instrument, such as spectrometers,^[^ ^65^ ^]^ microscopy,^[^ ^66^ ^]^ colorimeter,^[^ ^67^ ^]^ etc., and match their quantitation and multiplexing capability via innovative design. This approach allows smartphones to perform quantitative assessments and transmit geo‐tagged test results to the healthcare system. Thus, the integration of virus detection and smartphones offers a solution for clinical diagnosis that has a higher clinical impact than its laboratory counterparts.^[^ ^13^ ^]^ Those emerging smartphone‐based devices may not be more sensitive than the laboratory gold standard, but such handheld, modular and scalable multifunctional signal detection devices will provide a more accessible POCT platform for viral disease surveillance and management.

## 3 Smartphone‐Based Point‐of‐Care Testing for Virus Detection

### 3.1 Sample Processing and Fully Integrated Virus Diagnostics

Sample preparation is always a bottleneck in virus diagnostics because it involves a multistep process that is often resource‐ and time‐intensive. In the laboratory‐based virus tests, specimen collection, preparation, and analysis are performed by well‐trained professionals at different locations and time points, resulting in long turnaround times. To translate virus detection from laboratories to smartphone‐based POCT, integrating appropriate sample preparation methods for different clinical samples of blood, nasal swab, saliva, interstitial fluid (ISF), sweat, stool, or urine are essential.

Conventionally, sample preparation begins with sample collection with the vacutainer, syringe, and needle, nasal swab kits, saliva collection tubes, and urine collection cups.^[^ ^62^ ^]^ In POCT, capillary sampling is most often used to collect blood from finger or hill prick.^[^ ^68^ ^]^ In terms of technical innovation, integrating microneedle to capillary blood collection devices has been developed to streamline sample collection in a minimally invasive manner.^[^ ^69^, ^70^ ^]^ Recently, microneedle patches have been used as an alternative in ISF collection.^[^ ^71^, ^72^, ^73^ ^]^ A microneedle patch can be integrated seamlessly with POC devices (LOAD, 3D paper‐based devices) offering the simple, integrated, and potential sample collection format. To date, however, none of these systems have become commercially available, possibly due to safety issues arising from microneedle exposure, as well as the manufacturability and reproducibility of the devices. Nowadays, the testing of saliva, nasal swab, stool, urine, or tears becomes a more popular norm for disease surveillance, due to its patients’ comfort, convenience, and noninvasive collection such as throat swabs and absorption pads.^[^ ^74^ ^]^ Minimally invasive or noninvasive approaches minimize user intervention, which are crucial to achieving seamless flow from sample collection to integrated smartphone‐based POCT.

Sample loading and pretreatment strategies need to be integrated and coupled with molecular assays depending on the sample types and target concentration in a miniaturized, automated, and cost‐effective format.^[^ ^62^ ^]^ For people with respiratory viral infections, samples collected by nasal swabs in a relatively closed nasal cavity usually contain higher amounts of virus compared to those from blood, which is very conducive to virus detection. However, detection becomes much more difficult to use saliva, stool, or urine samples for diagnostics, in which the concentration of viral biomarkers is usually several thousand times lower than that in blood.^[^ ^74^, ^75^, ^76^ ^]^ Therefore, cell lysis, target separation or enrichment are often required to remove matrix fluids and interfering molecules in complex biological samples. To extract NAs from samples, chemical, mechanical, thermal, and electrical lysis are widely integrated into various types of POC devices, including microfluidic and acoustofluidic chips.^[^ ^74^ ^]^ The integration of microfluidic chips into smartphone‐based POC devices is a potential strategy to automate sample extraction, fluid transport, target concentration and detection. For example, LOAD systems have enabled plasma separating,^[^ ^77^ ^]^ immunoaffinity‐based bead capture,^[^ ^78^ ^]^ nucleic acids extraction and purification derived by a centrifugal force,^[^ ^55^ ^]^ fully automated sample preparation, molecular and protein assays in a smartphone‐based POC device.

Paper‐based devices is another approach for sample preparation and can be easily integrated into smartphone‐based POCT. Recently, paper‐based centrifugation has achieved plasma separation from a finger prick at milliliter‐scale sample volumes.^[^ ^79^ ^]^ Remarkably, the paper centrifuge reduces the infrastructure and cost barriers to whole blood processing in the field. To eliminate centrifugation steps in sample preparation, plasma separation membranes were mostly employed in lateral flow assays or 3D *μ*PADs.^[^ ^79^, ^80^, ^81^, ^82^ ^]^ Based on size exclusion, blood cells are trapped in the membrane while the plasma is separated and transmitted into the detection zones for detection. In comparison to microfluidic chips, those fully integrated paper‐based devices have the advantages of rapid reaction times, quick readout, ease of use, and low cost. The full integration of sample preparation, pre‐amplification (e.g., RCA, LAMP, CRISPR/Cas), and highly specific affinity molecule reactions with paper‐based assays that match with smartphone‐based POC devices are expected to achieve laboratory testing performance.

Signals transduction and data processing from a cartridge or single chip is essential for fully integrated smartphone‐based POC molecular diagnostic systems. Transducers capable of generating optical, electrical, thermal, magnetic, or acoustic signals corresponding to the target concentrations play a critical role in smartphone‐based POCT systems. The built‐in smartphone's camera can be used as optical detectors for colorimetric, fluorescent, or Raman signals, enabling smartphones as an instrumental interface for widely integration of various POCT chips or assays. For example, the development of self‐driven, smartphone‐controlled optical microfluidic chips enables automated sample preparation, nucleic acid amplification, and signal transduction through a customized cartridge. Furthermore, the wireless connectivity and telemedicine of smartphones, along with the customized applications, offer unique advantages in electrical sensors, allowing smartphones as a controller to automatically manipulate electrical POCT devices.^[^ ^83^, ^84^, ^85^ ^]^ In this regard, paper‐based electrical devices provide sensitivity, inexpensive methods, and can be directly integrated with smartphones via USB connectors, NFC, or Bluetooth. Despite great efforts have been made to develop smartphone‐based POCT systems, fully incorporating virus diagnostics into a device remains a challenge in system design, components, sample preparation, molecular assays, integration, and commercialization.

### 3.2 Smartphone‐Based Optical Biosensor

The smartphone‐based optical biosensors have many advantages due to their simple design and low cost. Generally, a qualitative test (e.g., yes/no) can be acquired using these devices by the naked eye or quantitatively via the use of an optical detector. Indeed, smartphones are poised to play a decisive role in the area of quantification by recognizing small differences in color tone via their cameras. According to the intensity of the color in test samples, the smartphone is capable of calculating the related concentration of the analyte based on the Beer–Lambert law.

#### 3.2.1 Smartphone‐Based Colorimetric Biosensors

Colorimetric assays measure the change in absorbance or reflection intensity of analyte complexes due to plasmon resonance or structural shifts in the optical characteristics of the sample. In colorimetric assays, smartphone cameras have been proven to identify and discriminate color tone variations.^[^ ^86^, ^87^, ^88^ ^]^ Bokelmann et al. reported a capture and improved LAMP (Cap‐iLAMP) assay for SARS‐CoV‐2 detection using a smartphone‐based color scoring system and an improved colorimetric RT‐LAMP assay.^[^ ^89^ ^]^ Cap‐iLAMP combines a hybridization capture‐based RNA‐extraction approach with RT‐LAMP to eliminate false positives. The color change induced by the addition of SYBR green I to RT‐LAMP products was captured using a smartphone camera, and the hue value was determined using the “Palette Cam” app. This POCT system allows the detection of single positive samples in pools of 25 negative samples in less than an hour, dramatically reducing the cost per test.

**Figure 6:**
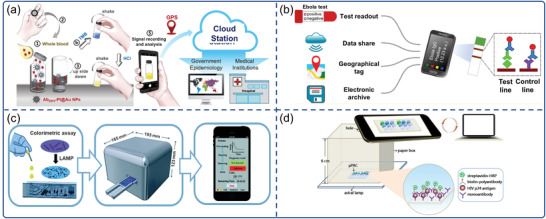
Smartphone‐based colorimetric biosensors for virus detection. a) The detection processes of the instrument‐free ZIKV POC test. Reproduced with permission.^[^ ^90^ ^]^ Copyright 2020, Elsevier. b) Smartphone combined with colloidal gold LFIAS for Ebola virus IgG detection. Reproduced with permission.^[^ ^93^ ^]^ Copyright 2018, American Chemical Society. c) A sample‐to‐answer, portable smartphone‐controlled system that integrated self‐driven LAMP microfluidic device for the detection of H1N1 virus. Reproduced with permission.^[^ ^98^ ^]^ Copyright 2019, Royal Society of Chemistry. d) Smartphone mediated paper‐based Dot ELISA system for HIV p24 antigen detection. Reproduced with permission.^[95]^ Copyright 2018, Elsevier.

Colorimetric immunoassays have been used extensively in viral disease diagnostics because of their simplicity. Recently, smartphone‐based POCT technology has integrated with colorimetric immunoassays to improve the performance of POC viral disease detection. For example, Hsu et al. described an immunosensor for point‐of‐care testing of Zika virus (ZIKV) with a high sensitivity of 1 pg mL^−1^ in an instrument‐free manner (**Figure** **6**a).^[^ ^90^ ^]^ In this accessory‐free system, the smartphone was used to photograph the colorimetric signal change, quantify the test results, and generate a graphical image of a surveillance map via a customized app, which simplified the instrumentation and improved infection management. Lateral flow immunoassay assays (LFIAS), the sample‐to‐answer POCT platforms, have been attracted much attention and widely used for the monitoring of virus disease outbreaks due to their cost‐effectiveness and simple operation.^[^ ^91^, ^92^ ^]^ Brangel et al. developed a serological platform consisting of in‐house colloidal gold LFIAS and a compatible smartphone reader for the semi‐quantification of Ebola‐specific antibodies within 15 min (Figure 6b).^[^ ^93^ ^]^ Three test lines plotted with three recombinant Ebola virus proteins and a control line were included on the test strip. A semi‐quantitative readout of the color intensity of the test zone of the strip was translated using a custom smartphone app. A smartphone was used to store, share, and geotag the data of the test subjects.

Over the past decade, microfluidic chips are considered an alternative to a centralized laboratory. Microfluidic chips can provide better analytical performance than conventional systems, due to their higher surface‐to‐volume ratio, faster rate of mass and heat transfer.^[^ ^6^ ^]^ Besides, these devices need lower consumption of patient samples, make an easier operation, and have higher reproducibility in the detection of pathogens.^[^ ^94^ ^]^ Featured in the automated bio‐analytical process, microfluidic chips are adequate for smartphone‐based miniaturized devices due to their much smaller size and more flexible design.^[^ ^95^ ^]^ At present, polymethyl methacrylate (PMMA),^[^ ^96^, ^97^ ^]^ polydimethylsiloxane (PDMS),^[^ ^98^, ^99^ ^]^ and filter paper are extensively used for the fabrication of microfluidic and microarray chips for biochemical analysis.^[^ ^100^ ^]^ Recent advances in NAATs, microfluidic chips, and smartphones enable portable detection systems for POC diagnosis. For example, a portable smartphone‐controlled system was developed and integrated with a passive, self‐driven LAMP microfluidic device for influenza A (H1N1) virus detection (Figure 6c).^[^ ^98^ ^]^ The system consists of a portable control device, a microfluidic chip and a smartphone. The portable control device featured stepping motors, neodymium magnet, photo‐interrupter modules, thermal control module, temperature sensor, punching‐press mechanism, color sensor and Bluetooth. The microfluidic chip consisted of a sample pretreatment module and a LAMP reaction module, and all required reagents were preloaded on the chip. The color sensor equipped in the portable control device could detect the colorimetric results of the on‐chip LAMP assay which could be wirelessly transmitted to and displayed on the smartphone via the customized app. The system was capable of purifying, lysing viral pathogens and executing isothermal nucleic acid amplification, as well as quantifying the results of colorimetric assays in 40 min.

μPADs are a competent system for affordable POC diagnosis. Using 2D networks of cut paper or 3D stacked patterned paper,^[^ ^101^, ^102^ ^]^ μPADs allow complex fluid handling without the need for external power. Li et al. demonstrated a portable immuno‐testing platform based on a smartphone and plastic micro‐pit array chips (*μ*PACs) for HIV p24 antigen detection (Figure 6d).^[^ ^95^ ^]^ The sandwich immunoassay on the *μ*PACs was captured by a smartphone camera and analyzed with ImageJ software. Despite the low‐cost and easy operation of *μ*PACs, user error and environmental interference of this system were unavoidable during the interpretation of test results in practical applications. To address this issue, Wu et al. described a smartphone‐based microfluidic Dot‐ELISA assay for influenza A detection.^[^ ^103^ ^]^ The whole microfluidic chip includes micro‐pumps, reagent storage, and reaction modules to enable integration and automation of the bio‐analytical process, which was controlled by a smartphone via Bluetooth. The image of paper‐based Dot‐ELISA in the middle of the reaction module was captured by a smartphone camera and displayed by a customized app. The color intensity of the images of testing dots was analyzed by an intelligent algorithm for semi‐quantitative detection. Notably, the tailored smartphone app was developed using Java language, can direct the microcontroller, capture and analyze the image, display, and transmit the detection result. CD4+ T cell testing is a critical parameter in monitoring the immunologic function of HIV‐infected patients.^[^ ^104^ ^]^ Kanakasabapathy et al. designed a smartphone‐based sensing system for CD4 testing which consisted of a smartphone accessory and a disposable microfluidic chip. The microfluidic chip was designed using CAD software and fabricated using a cut PMMA and a modified glass.^[^ ^96^ ^]^ The sample was added into the modified microfluidic chip through the inlet, and then CD4+ T cell was captured by immobile anti‐human CD4 antibodies. After capture, the microfluidic device was inserted into the optical setup to count CD4+ T‐cells using the rear camera of a smartphone. The smartphone app was developed to capture images of the sample and use an adaptive thresholding algorithm to determine the number of cells.

Laboratory‐based ELISA usually requires complex instruments such as microplate readers with a time‐consuming process. To address these shortcomings, Laksanasopin et al. developed a complete laboratory‐quality immunoassay platform, the “smartphone dongle,”^[^ ^105^ ^]^ where almost all the optical, mechanical, and electronic functions of a laboratory‐based ELISA could be replicated by accessing a smartphone and with no stored energy. The “dongle” with a microfluidic disposable cassette was attached to a smartphone, enabling a power‐free vacuum generator via power from the audio jack connector, and frequency shift keying (FSK) data transmission to the phone via the audio jack. A microcontroller was programmed to perform FSK transmission by converting the photodiode readings. A microfluidic cassette containing reagent and test cassettes was used to automatically perform a multiplexed immunoassay, and the optical density (OD) (absorbance) of silver enhancement on each assay was transmitted and recorded using a touch‐activated pictorial smartphone dongle app. In a blinded experiment, the smartphone dongle exhibited a sensitivity of 92% and 100% for HIV and syphilis, respectively. This microfluidic‐based smartphone dongle was also demonstrated for simultaneous detection of hemoglobin and HIV antibodies by Guo et al., suggesting the platform was accessible to smartphones users.^[^ ^106^ ^]^ In another work, Thiha et al. designed a Lab‐on‐Compact Disc (LOCD) platform to measure the absorbance using a monochromatic light source and to interpret the ELISA test results using photo sensor circuitry.^[^ ^107^ ^]^ The absorbance values from the test results were transmitted via Bluetooth to a smartphone. This LOCD platform was demonstrated a high degree of accuracy in the detection of antibody IgG in 64 dengue patients.

#### 3.2.2 Smartphone‐Based Fluorescence Biosensors

##### Smartphone‐Based Fluorescence LFIAS Sensors

**Figure 7:**
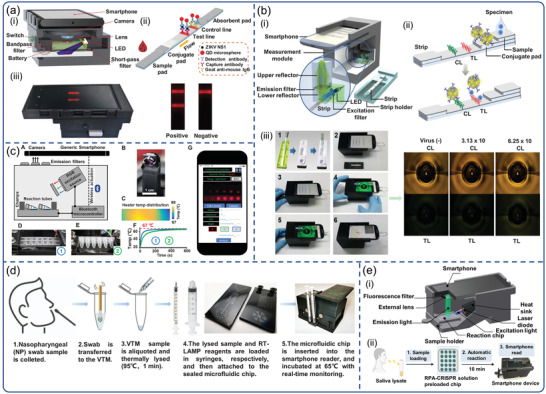
Smartphone‐based fluorescence biosensors for virus detection. a) The design of the smartphone‐based fluorescent LFIAS platform for the detection of ZIKV NS1. Reproduced with permission.^[^ ^113^ ^]^ Copyright 2019, Elsevier. b) Overview of smartphone‐based fluorescence LFIAS reader design. Smartphone‐based LFIAS reader including a reflective light concentrator module, a fluorescence detector flow strip, and the test procedures of the purposedplatform. Reproduced with permission.^[^ ^114^ ^]^ Copyright 2016, Ivyspring International Publisher. c) Smartphone‐enabled LAMP box for analysis of Zika viruses with RT‐LAMP assay. Reproduced with permission.^[^ ^124^ ^]^ Copyright 2017, Nature Publishing Group. d) Workflow for the detection of SARS‐CoV‐2 using smartphone‐based imaging POC system. Reproduced with permission.^[^ ^125^ ^]^ Copyright 2020, National Academy of Sciences, USA. e) Schematic of a 3D‐printed smartphone fluorescence reader, and workflow of a saliva‐based on‐chip CRISPR‐FDS smartphone assay. Reproduced with permission.^[^ ^135^ ^]^ Copyright 2020, American Association for the Advancement of Science (AAAS).

Fluorescence‐based assays are one of the most widely used optical detection methods due to their high sensitivity,^[^ ^108^ ^]^ good anti‐interference, and robustness,^[^ ^109^ ^]^ fast signal speed,^[^ ^110^ ^]^ and simple operation.^[^ ^111^ ^]^ Fluorescent LFIAS sensors, which have a higher sensitivity and accuracy compared with traditional LFIAS sensors based on gold nanoparticles (AuNPs) or colored latex nanoparticles,^[^ ^112^ ^]^ have attracted great attention in quantitative detection of viral diseases.^[^ ^91^ ^]^ Recently, the smartphone‐based LFIAS reader enabled image capture and processing, as well as quantitative calculation of fluorescent areas in test results via a customized app algorithm. For instance, Rong et al. developed a smartphone‐based fluorescent quantum dots (QDs)‐LFIAS platform for the detection of Zika virus nonstructural protein 1 (NS1) (**Figure** **7**a).^[^ ^113^ ^]^ The smartphone‐based fluorescent LFIAS platform integrated a 3D‐printed accessory, an external optical (UV LED, 5 W, 365 nm), a bandpass filter (624/40 nm), a short‐pass filter (425 nm), and electrical components with a smartphone. The excitation light was designed to illuminate the LFIA strips at a 60° incidence angle, which significantly reduced background noise. In addition, the plano‐convex lens and bandpass filter fixed in front of the smartphone camera ensured the acquisition of high‐quality fluorescence images. Using ImageJ software, this system quantified the ZIKV NS1 with a sensitivity of 0.15 ng mL^−1^ within 20 min. Yeo et al. designed a smartphone‐based fluorescent strip reader for the detection of three different AIV subtypes (H5N3, H7N1, and H9N2) by exploiting an efficient reflective concentrator.^[^ ^114^ ^]^ The utilization of refractive optics technique overcame the limited numerical aperture of the smartphone‐based LFIAS reader (Figure 7b). The LED was used as excitation light powered by a smartphone via universal serial bus port, requiring no additional battery. The images of the fluorescence intensities of LFIAS were captured with a smartphone camera, and the ratio between the test and control lines was calculated using an app. Moreover, the results from distributed smartphones could be wirelessly transmitted and collected by a centralized database system via a short messaging service (SMS), along with the measurement time and location. The smartphone‐based diagnostic system showed a total coincidence rate of 96.55% (95% confidence interval [CI]) in clinically confirmed H5N1 patients with an optimized bioconjugate. Recently, Yeo et al. achieved rapid and simultaneous detection of influenza A and H5 subtypes based on QDs‐LFIAS using the same platform.^[^ ^115^ ^]^ Differently, two independent emission filters (580 and 650 nm) were inserted into the reader to obtain two separate fluorescent signals from QDs in the test lines

Upconversion nanoparticles (UCNPs) have become attractive nanomaterials in the development of UCNP‐LFIAS due to their long‐term photostability and low signal‐to‐noise ratio. Gong et al. developed a miniaturized and portable lateral flow assay (LFA) detection system, including a smartphone‐based UCNP‐LFA analyzer and a custom‐designed app.^[^ ^116^ ^]^ LFA analyzer consisted of a compact infrared laser, a dichroic mirror, and an infrared filter. The laser horizontal plane excitation light transmitted through the dichroic mirror at 45°, excited UCNPs on the LFIAS, reflected visible luminescence, and effectively eliminated stray light. The fluorescent intensity of UCNPs on test and control lines was captured using a smartphone camera and then converted to the sample concentration via built‐in RGB value algorithms in the UCNP‐LFA app. This LFA detection system showed great potential in quantitative analysis of nucleic acid (hepatitis B virus and HBV) in a real‐time manner.

##### Smartphone‐Based Nucleic Acid Amplification Platforms

Typically, nucleic acid‐based tests utilize an enzymatic amplification process to millionfold amplify target sequences. Nowadays, NAATs have demonstrated significant advantages in detecting and controlling newly discovered or emerging viral pathogens. Ahrberg et al. developed a smartphone‐sized real‐time PCR device for Ebola virus RNA detection.^[^ ^117^ ^]^ This system consisted of miniaturized fluorescence housing, reverse transcription PCR (RT‐PCR), and melting curve analysis (MCA), which was conducted in less than 37 min. LEDs were used for stimulation and photodiodes were used to detect the fluorescence emitted in the RT‐PCR reaction chamber. To process the photocurrent signals, a lock‐in amplifier was embedded, significantly reducing background noise. ZIKV has been a major global public health concern due to its association with fetal microcephaly and Guillain–Barré Syndrome (GBS).^[^ ^118^, ^119^ ^]^ Chan et al. described a portable molecular diagnostics system for ZIKV infection which could automatically perform reverse transcription recombinase polymerase amplification (RT‐RPA) on up to 12 samples within 15 min.^[^ ^120^ ^]^ Subsequently, the fluorescent image from probe‐based RT‐RPA assays was taken by a smartphone camera for further analysis with simple operation and minimal device requirements.

Isothermal nucleic acid amplification meets the criterion of a sample‐in, answer‐out device.^[^ ^121^ ^]^ For example, LAMP eliminates the need for thermocycling and simplifies the complexity of equipment while maintaining satisfactory sensitivity or specificity in clinical samples.^[^ ^122^, ^123^ ^]^ Priye et al. combined a smartphone‐enabled LAMP box with RT‐LAMP assay for simultaneous analysis of Zika, dengue, and chikungunya viruses.^[^ ^124^ ^]^ As illustrated in Figure 7c, the NAATs device consisted of three modules: a heating module, an assay reaction housing module, and an optical image‐analysis module that was controlled via Bluetooth by a custom smartphone app. Notably, the application introduced a new chromaticity algorithm that analyzed multiplexed RT‐LAMP detection fluorescence images taken by the smartphone camera based on color and brightness. For the detection of the ZIKV, the algorithm demonstrated higher accuracy in distinguishing positive and negative signals from human samples (blood, urine, and saliva). In another study, instead of RGB intensities, Priye et al. utilized the CIE xyY (chromaticity‐luminance) color space for measuring the luminance of the fluorescent signals generated by target nucleic acid amplification, and designed a new smartphone‐based image analysis pipeline.^[^ ^88^ ^]^ This chromaticity‐luminance assay achieved simultaneous detection of Zika and chikungunya viral RNA via RT‐LAMP assay.

Notably, most of the reported platforms required complicated sample preparation progress, such as nucleic acid extraction and separation, that add complexity and cost. Recently, Ganguli et al. employed a complete hands‐free sample processing microchip,^[^ ^125^ ^]^ a unique RT‐LAMP, and smartphone‐based imaging POC system for detection of SARS‐CoV‐2 (Figure 7d). The imaging system was composed of a portable 3D‐printed cradle that assembled optical and electrical components, a smartphone, a thermal control module, and a rear‐facing smartphone camera interface. The swab was immersed in viral transport medium (VTM) and thermally lysed (95 °C, 1 min), which was further mixed with RT‐LAMP reagents for rapid, reliable, and automated amplification in the amplification chip in 30 min. Real‐time monitoring of fluorescence intensity of RT‐LAMP data was achieved using a smartphone‐based reader. However, this platform relied on optical components (e.g., excitation light and optical filters), which increased the background signals. Moreover, the only use of smartphone cameras to image did not take advantage of connectivity due to the lack of smartphone‐based image quantitative processing. Song et al. fabricated a smartphone‐based PMMA‐microchip platform for quantitative detection of ZIKV and HIV.^[^ ^97^ ^]^ The microchip platform combined bioluminescent assay in real‐time and LAMP technology (BART‐LAMP) with smartphone‐based detection. The device consists of a thermos cup body, a 3D‐printed holder, a smartphone, and a chip with four reactors for on‐chip NA extraction and BART‐LAMP assay. The customized smartphone app was designed to real‐time image/record the bioluminescence signal, process images, quantify nucleic acid concentration, and report/transmit test results. This inexpensive, hand‐held smartphone‐connected cup (SCC) eliminates the need for excitation sources and optical filters, simplified sample preparation progress, and provided a rapid, connected and quantitative detection platform for ZIKV in urine and HIV in blood.

##### Smartphone‐Based Digital PCR Platforms

Rapid POC quantification of low virus RNA or DNA load would significantly reduce the turnaround time for virus detection and timely curb the spread of epidemic. Digital PCR (dPCR) holds the promise of high sensitivity and accuracy in the absolute quantification of nucleic acids.^[^ ^126^, ^127^, ^128^ ^]^ In dPCR, nucleic acids samples are distributed to tens of thousands of unique reaction vessels or microdroplets at a single nucleic acid level, which enriches the virus nucleic acids and reduces the influences of PCR inhibitors^[^ ^129^ ^]^ To date, commercial dPCR products are mainly divided into microchamber‐based dPCR (for example, Fluidigm Biomark system, QuantStudio 3D system) and droplet‐based dPCR (BioRad QX200 system, Raindance RainDrop system).^[^ ^130^, ^131^ ^]^ However, these commercial dPCR platforms typically rely on multiple independent units to complete the sample dispersion, on‐chip amplification, and digital detection, resulting in complex and bulky overall system that is unaffordable in resource‐limited areas. To address this issue, Hu et al. reported a smartphone‐based droplet digital LAMP device for quantification of low abundance cfDNA and gene mutation.^[^ ^132^ ^]^ The microchip integrated immiscible phase filtration, reagent mixing, droplet generation, and digital LAMP amplification. Fluorescent images from the droplet digital LAMP chip were captured by a smartphone camera and further analyzed. However, this system suffered from complicated manual operations and manual intervention. Gou et al. developed a fully integrated smartphone‐based dPCR sensing system for highly accurate DNA quantitative analysis.^[^ ^130^ ^]^ The dPCR sensing system integrated a miniaturized thermocycler, an on‐chip dPCR into a smartphone‐based optical device, and the whole integrated function units were automatically controlled by a custom Android software. Recently, Cao et al. reported a droplet digital LAMP platform based on a microgel array chip and hand‐held smartphone‐based reader.^[^ ^133^ ^]^ The microgel array chip held the capability of “sample self‐absorption and partition” with thousands of isolated microgels, avoiding professional operations and auxiliary equipment. Although there are few reports of smartphone‐based dPCR systems for virus screening, it is expected to become an enormous potential platform for POC nucleic acids quantitation analysis in resource‐limited settings.

##### Smartphone‐Based CRISPR Technology Platforms

The requirement for NAATs that are fast, widespread, and capable of obtaining ultrasensitive diagnostic results has prompted efforts to explore new strategies. To reduce the required expertise and infrastructure, isothermal preamplification of target RNA, such as RPA, is a commonly used strategy. Recently, by combining RPA with Cas12a and Cas13 proteins, the DETECTR and SHERLOCK systems were developed, which enable the POC detection of nucleic acids with high sensitivity, specificity, and reliability.^[^ ^134^ ^]^ Ning et al. developed a smartphone‐read CRISPR assay with a 15‐min sample‐to‐answer time for saliva‐based POC SARS‐CoV‐2 diagnosis.^[^ ^135^ ^]^ The smartphone‐based fluorescence microscope device integrated a laser diode, a lens filter, a chip slot, a smartphone socket, a power switch, and an emission filter for the smartphone camera (Figure 7e). On an assay chip, the swab sample was lysed with lysis buffer and mixed with RT‐RPA and CRISPR‐Cas12a. After inserting the chip into the smartphone reader, the fluorescence images of the assay chip were captured by a smartphone camera and analyzed using ImageJ software. This smartphone‐readable CRISPR assay offers the potential to rapidly expand SARS‐CoV‐2 diagnosis in resource‐limited screening sites. However, this platform requires pre‐amplification of the viral genome and subsequent addition to the assay chip for analysis, adding to the potential for variation and error, as well as operational complexity. Recently, Fozouni et al. reported an amplification‐free CRISPR‐Cas13a assay for SARS‐CoV‐2 screening by using a smartphone‐based microscope. The mechanism relies on the increased Cas13a activation by multiple crRNAs.^[^ ^134^ ^]^ This portable diagnostic device included low‐cost collection optics and laser illumination. The change in fluorescence over time after cleavage by active Cas13 was monitored by the compact smartphone‐based microscope. Offline image time series analysis was performed using a custom MATLAB (Mathworks) script. The platform directly quantified viral load of preisolated SARS‐CoV‐2 RNA and exhibited a sensitivity of 100 copies mL^−1^ in 30 min, indicating a consumer electronic‐based diagnostic device rather than a specialized instrument in central laboratory.

##### Smartphone‐Based Fluorescence Microarray Platforms

Droplet‐based digital bioassays are thought to be a highly sensitive and high‐throughput analytical approach for POC diagnosis. Natesan et al. reported a digital and multiplexed microarray platform for telemonitoring Ebola and Marburg filovirus infections.^[^ ^136^ ^]^ A flow cell microarray and a smartphone fluorescence reader were used to recognize recombinant antigens from six filoviruses. The smartphone was connected to the opto‐electro‐mechanical hardware and preinstalled with customized smartphone software, acting as a fluorescence reader, controlling operation, acquiring, and distributing test results via cloud service. Minagawa et al. developed a smartphone‐based imaging platform to obtain the neuraminidase activity of influenza viruses by measuring the fluorescence of droplets, thus enabling influenza virus digital counting.^[^ ^137^ ^]^ The platform assembled the smartphone with other optical components, including a power LED, a micrometer head, an aspherical lens, a long pass filter, and a femtoliter reactor array device (FRAD). The FRAD was illuminated by a power LED which was activated via a smartphone app. The fluorescence images captured by the smartphone camera were saved and further analyzed using ImageJ software. Sensitivity of the digital smartphone counting device was 100 times greater than that of a commercial influenza diagnostic kit. Similarly, Jiang et al. utilized catalytic hairpin assembly (CHA) amplification reactions and a smartphone camera achieved simultaneously detecting avian influenza virus (H1N1, H7N9, and H5N1) within 20 min.^[^ ^138^ ^]^ The smartphone was used to capture fluorescence images of the sample spot on the microarray, which were then analyzed at the grey level for quantifying the viral load.

### 3.3 Smartphone‐Based Electrochemical Biosensor

Owing to its simplicity, reliability, and cost‐effectiveness, electrochemistry has demonstrated broad applications for quantitative detection of important analytes in clinical diagnostics. More importantly, electrochemical methods can be easily implemented in miniaturized and portable instruments and have a large range of applications for in situ POCT‐based assays.^[^ ^139^, ^140^ ^]^ On this basis, smartphones are gradually integrated with such portable instruments to become an important part of controlling, recording, and displaying electrochemical detection, thus realizing smartphone‐based electrochemical sensing platforms for POCT.^[^ ^141^, ^142^ ^]^

#### 3.3.1 Smartphone‐Based Voltammetric POCT

**Figure 8:**
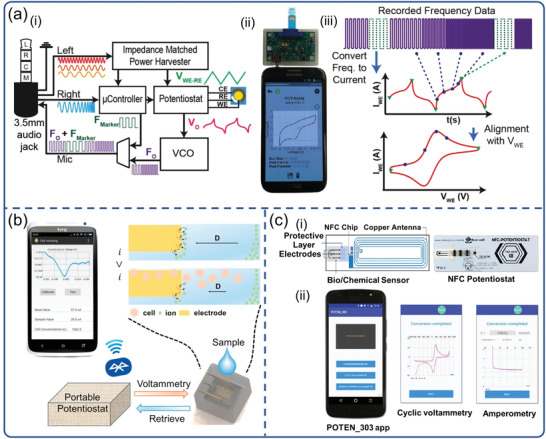
Smartphone‐based electrochemical biosensors for virus detection. a) POC smartphone‐based potentiostat platform with CV to detect HCV. Reproduced with permission.^[^ ^143^ ^]^ Copyright 2016, Elsevier. b) System diagram and electrochemical principle for WBC count with DPV. Reproduced with permission.^[^ ^145^ ^]^ Copyright 2017, Elsevier. c) The SIC4341 circuit board diagram and the image of portable NFC Potentiostat, and the interface of POTEN_303 app. Reproduced with permission.^[^ ^147^ ^]^ Copyright 2021, Elsevier.

Voltammetry is an electrochemical technique based on redox reactions and widely used to study the kinetics and thermodynamics of electron transfer in chemical reactions. The information of the analyte is obtained from the register of the resulting electric current that appears on the working electrode as a function of the applied potential. Aronoff‐Spencer et al. reported a smartphone‐based potentiostat which can implement cyclic voltammetry (CV) for Hepatitis C virus (HCV) determination (**Figure** **8**a). In that study, a dual‐affinity biobrick chimera was developed as the reporter by conjugating gold binding peptide (GBP) to the yeast cell lines which has been genetically engineered to present surface HCV core antigen.^[^ ^143^ ^]^ The system was composed of a radiochemical device and a smartphone, with a headphone jack on the smartphone for signal transmission. However, the AC‐coupled audio channels present the main challenge with using a headphone jack, because no DC signal or power can be directly transmitted between the phone and the potentiostat. Fortunately, such challenge can be overcame by voltage‐controlled oscillator (VCO), since VCO is able to convert the DC signal output by the potentiostat to a frequency signal within the audio band which then can be sent to a smartphone for recording, demodulation, and reconstruction. To make it easier for smartphones to reconstruct voltammograms, that is, the relationship between the current and the input voltage measured by CV, the microcontroller generated marker tones representing the voltage transition point of the applied potential, and inserted them into the output of the VCO as the data was processed. This smartphone‐based system is highly consistent with commercial electrochemical workstations, resulting in a more affordable approach for POC diagnostics. Subsequently, João et al. also reported a similar method for HCV detection, with excellent sensitivity and a lower cost of about $0.2.^[^ ^144^ ^]^

In addition, portable electrochemical devices based on differential pulse voltammetry (DPV) are also designed and manufactured for the detection of various viruses. For instance, Wang et al. reported a smartphone‐based DPV portable device, which enables indirect detection of viruses by counting white blood cells (WBCs) (Figure 8b).^[^ ^145^ ^]^ WBCs analysis provides rich information in both rapid diagnosis of acute infection and chronic disease management. POC applications were highlighted by implementing a handheld smartphone DPV measurement system via wireless Bluetooth communication. Because the absorption of WBCs on the electrode surface would prevent the electron transfer, the DPV device could easily detect the content of WBCs. Beduk et al. reported a portable POC electrochemical analyzer (KAUSTat) connected to a smartphone to detect SARS‐CoV‐2 S1 spike protein.^[^ ^146^ ^]^ A miniaturized electrochemical immunosensor based on laser‐scribed graphene (LSG) was integrated into a homemade KAUSTat analyzer and operated using KAUSTat software. KAUSTat consisted of built‐in memory, a connectable add‐on device, a slot for an SD card, a battery, Bluetooth and a mini‐USB connector, enabling multichannel measurements. Using electrochemical immunoassay and KAUSTat analyzer, the accurate detection of S‐protein was achieved by detecting signal changes and recording by DPV method within only 1 h of incubation. It is worth noting that the DPV method is very similar to the CV method, and both belong to the category of potential sweep methods. However, DPV is more accurate because it reconstructs, encodes, and records the signal after subtracting the Faraday background current from the generated signal. Thus it can greatly reduce background interference and improve detection sensitivity.

#### 3.3.2 Smartphone‐Based Amperometric POCT

Amperometric biosensors are designed to provide quantitative analytical information of an electroactive biological target by measuring the current generated by its oxidation or reduction. The introduction of smartphones can help achieve more functional and portable POCT systems. Teengam et al. presented a compact smartphone‐controlled amperometric biosensor for quantitative detection of HBV (Figure 8c).^[^ ^147^ ^]^ The completed system combined a card‐sized electrochemical near field communication (NFC) sensor with a smartphone to control and receive signals from immunoassay. Amperometric measurements were carried out by a POTEN 303 app that commanded the measurement parameters, received data in real‐time and displayed results via NFC on smartphone screen. The cost for a set of NFC potentiostat system is <$10, exhibiting the potential to address the challenges in terms of test cost and improving patient outcomes. Similarly, Nemiroski el at. reported a universal mobile electrochemical detector for the detection of malaria using chronoamperometry.^[^ ^148^ ^]^ It was connected to a radiochemical device and a smartphone via the audio channel. Also, the smartphone‐based electrochemical device was capable of performing all of the most common electroanalytical techniques, such as potentiometry, CV, DPV, and square wave voltammetry. The universal compatibility of smartphones ensures that complex diagnostic testing can be performed by end‐points with a diverse range of needs and resources, and that regional disease control efforts can be informed.

#### 3.3.3 Smartphone‐Based Impedimetric Biosensors

Impedance‐based biosensors work according to the principle of electrical impedance and are constructed by attaching a biometric element to the electrode surface. The target analyte is detected by measuring and/or monitoring the electrical impedance signal to obtain a signal proportional to the analyte activity. The introduction of smartphones can help achieve more functional and portable POCT systems. Kaushik et al. presented an immunosensor for ZIKV‐protein detection with high sensitivity and specificity.^[^ ^47^ ^]^ The miniaturized immunosensor was fabricated by functionalizing interdigitated micro‐electrode of gold (IDE‐Au) array with ZIKV specific envelop protein antibody (Zev‐Abs). The electrical response of the immunosensor which was a function of ZIKV protein concentrations, was measured by performing electrochemical impedance spectroscopy (EIS). Integrated this ZIKV‐protein specific immunosensor with a smartphone‐controlled miniaturized potentiostat (MP)‐interfaced is promising for ZIKV infection diagnosis in low resource setting.

### 3.4 Other Types of Sensors

**Figure 9:**
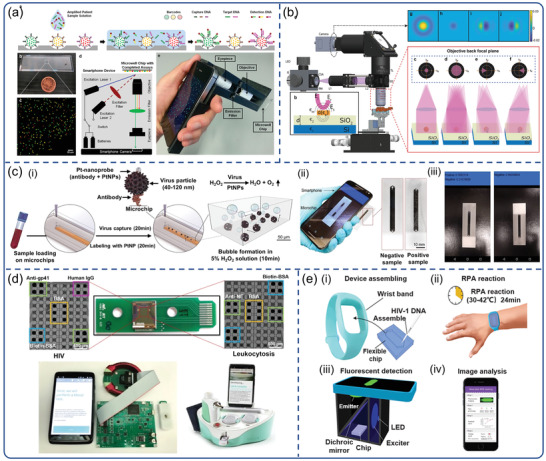
a,b) Smartphone‐based microscopy for virus disease diagnostics. a) Overview of the smartphone‐based microscopy utilizing quantum dot barcodes. The assay involves the addition of patient samples to a chip coated with microbeads and related optical principles. Reproduced with permission.^[^ ^149^ ^]^ Copyright 2015, American Chemical Society. b) SPIR microscopy and schematic of the experimental setup. Reproduced with permission.^[^ ^150^ ^]^ Copyright 2018, American Chemical Society. c–e) Smartphone integrated with other types of biosensors for virus disease diagnostics. c) 3D schematic of the CNN‐nanoparticle‐enabled smartphone for virus detection. Reproduced with permission.^[^ ^152^ ^]^ Copyright 2020, American Association for the Advancement of Science (AAAS). d) Image of GMR nanosensor chip on PCB, and essential components of the redesigned Eigen diagnosis platform. Reproduced with permission.^[^ ^153^ ^]^ Copyright 2019, Elsevier. e) Wearable RPA sensing system for rapid detection of HIV‐1 DNA is consisted of a fluorescence image box and PDMS‐based flexible chips. Reproduced with permission.^[^ ^155^ ^]^ Copyright 2019, Elsevier.

In principle, a smartphone camera can replace laboratory‐based spectrometers, and match related quantitation and multiplexing capability. Ming et al.^[115]^ developed a chip‐based wireless diagnostic device by integrating QD barcode technology and isothermal amplification with smartphone‐based microscopy (**Figure** **9**a).^[^ ^149^ ^]^ The handheld device is assembled with laser diodes, lenses, filters, and 3D‐printed plastic chassis batteries. The two laser diodes are switched on independently via a switch, allowing for simultaneous detection of multiple biomarkers. The moveable objective lens magnifies and the eyepiece focuses QD barcodes, allowing them clearly visible on the smartphone camera. Moreover, the use of multiple filters increases the accuracy of barcode discrimination, and the analysis of these images was also achieved by a custom‐specific algorithm. This device enables the rapid diagnosis of infections in HIV or hepatitis B patients in less than an hour with a LOD of 1000 viral copies per milliliter in a single test. However, conventional imaging techniques still face some limitations, such as the low difference in refractive index and limited spatial resolution. Recently, Yurdakul et al. reported a wide‐field single‐particle interferometric smartphone microscopy (SR‐IRIS), enabling high‐throughput visual discrimination in morphology of a diverse population of Ebola virus‐like particles and Ebola vaccine candidates (Figure 9b).^[^ ^150^ ^]^ Because the SR‐IRIS utilizes SiO_2_ layered substrate in an interferometry configuration, constructive reference field is allowed through specular reflection. A narrow‐band light source, that is, LED, achieved interferometric detection and provided an order of magnitude of coherence length greater than the layer thickness. Furthermore, asymmetric illumination and efficient computational algorithms were applied to the captured images for the improvement of the lateral resolution of SPIR microscopy. This smartphone‐based microscopy demonstrated a twofold lateral resolution improvement over a large field‐of‐view at a resolution of ∼150 nm with no extra labels and sample preparation.

Biological samples that illustrate changes often generate large amounts of data, so computational and statistical methods such as deep learning, the Internet of Things (IoT), and other artificial intelligence (AI) methods can be used for diagnosis.^[^ ^151^ ^]^ AI can also be employed during the outbreak of the epidemic for the development of new POC strategies. For example, Draz and coworkers developed a convolutional neural network (CNN)‐based smartphone system for rapid and sensitive detection of HBV, HCV, and ZIKV (Figure 9c).^[^ ^152^ ^]^ To facilitate sample testing, a custom‐built microfluidic cartridge utilizing catalytic properties of platinum nanoparticles (PtNPs) was developed to generate gas bubbles that create patterns for virus detection on‐chip. The nanoparticle‐enabled smartphone (NES) system is consisted of an Android smartphone equipped with a trained CNN algorithm capable of imaging and analyzing bubbles in a straight microchannel. The system simplifies sample processing and is adaptable to various smartphone models without the need for external optical hardware for signal detection, resulting in a powerful universal modality for low‐cost POC diagnosis.

In a recent example, Ng et al. demonstrated a smartphone‐based magneto‐nanosensor platform that utilized magneto‐immune‐nanosensor arrays and magnetic nanoparticles (MNPs) for detection of HIV in saliva (Figure 9d).^[^ ^153^ ^]^ The giant magnetoresistive (GMR) nanosensor platform included a disposable cartridge, a processor station (PCB), and a smartphone. The magneto‐nanosensor chip, employing the GMR effect, consisted of an array of magnetic sensors which could monitor the change in electrical resistance induced by the binding of MNPs. The platform completed circuitry, signal processing and user‐interface application, simplifying operation, and reducing electrical noise, thus facilitating self‐testing in the POC setting. Bioluminescence is a powerful analytical method because of its low background and simple instrumentation, which has been widely used in biomedical imaging, small molecule analysis, disease prevention, and food safety control. Arts et al. developed a sensor platform (LUMABS) for antibodies detection using bioluminescence resonance energy transfer (BRET) via a smartphone.^[^ ^154^ ^]^ Using only the smartphone camera as the equipment, this LUMABS allowed to detect antibody concentrations against hemagglutinin (HA), HIV1‐p17, and dengue virus type I in blood plasma at the picomolar level.

Wearable devices as powerful tools for personal healthcare monitoring have greatly attracted the interest of researchers in monitoring physiological parameters and biomarkers. Kong et al. developed a wearable microfluidic device that integrated RPA assay into a smartphone fluorescent detection system for simple and rapid HIV‐1 DNA detection (Figure 9e).^[^ ^155^ ^]^ The RPA reactions in the flexible chip were processed via human body heat and recorded by a smartphone‐based fluorescence detection system. The fluorescence signal from RPA reactions was excited by the excitation light and filtered by an emission filter, eventually captured by the smartphone camera and analyzed using ImageJ software. This wearable microfluidic sensor combined with a smartphone‐based fluorescence detection system was able to detect HIV‐1 DNA at 100 copies mL^−1^ in 24 min.

Technological advances in POCT have made it possible to use a smartphones as wireless or connected instrumental interface with POCT devices equipped with signal processing units. While POC virus diagnostics are booming, however, signal transducers in smartphone‐based POCT have yet to be fully exploited. For instance, surface acoustic wave (SAW) biosensor has been widely reported for various virus detection such as Ebola virus,^[^ ^156^ ^]^ influenza virus,^[^ ^157^ ^]^ dengue virus,^[^ ^158^ ^]^ because of their high sensitivity, rapidness, small sizes, and remote accessibility. However, few SAW devices have already been successfully integrated into smartphone‐based devices for POCT applications. One important reason is that some limitations have not been effectively solved, including damping effect in solution, high packaging costs, and difficulty in integrating with chips.^[^ ^159^ ^]^ Recently, Turbe et al. developed a smartphone‐connected SAW device for HIV detection.^[^ ^160^ ^]^ A SAW biochip was connected to a pocket‐sized control box reader and a smartphone. The SAW biochip sensitively detected the phase changes of shear horizontal surface acoustic wave (SH‐SAW) caused by the immune binding of HIV and the specific antibodies, and then sent the signals to the smartphone via Bluetooth. A user‐friendly smartphone app enables the analysis, display, and transfer of results.

As another form of acoustic wave biosensors, quartz crystal microbalance (QCM) based biosensors have received great interest due to their high sensitivity and label‐free strategy. The QCM is based on the piezoelectric effect, whose resonant frequency is influenced by the mass per unit area at the crystal surface, and thus able to detect virtually any type of biomolecule.^[^ ^161^, ^162^ ^]^ Excitingly, great efforts have been spent on developing QCM‐based devices for early virus diagnosis,^[^ ^163^, ^164^, ^165^, ^166^ ^]^ making it possible to integrate with smartphones for the detection of various viruses in the future.

Thermal sensor is another type of signal transducers which has not fully exploited to be integrated with smartphone‐based sensing systems. In comparison to acoustic wave biosensors, thermal sensors are easy to be integrated and implemented in POCT devices, since no complicated electronic devices and signal processing units will be needed. Recently, emerging thermal imagers have become commercially available at a low cost, allowing thermal sensors to be applied on smartphones.^[^ ^167^, ^168^ ^]^ For example, it has been demonstrated to connect a commercial thermal imager to a smartphone camera for exosome detection based on Au@Pd nanoparticles and aptamer‐nanoflower‐assisted thermal LFS (ANAN‐LFS).^[^ ^169^ ^]^ Recently, Guo et al. utilized a thermal imaging sensor connecting with a smartphone via a USB port to read the signals in a microfluidic chip, and successfully detected Salmonella with a LOD as low as 93 CFU mL^−1^ within 1 h.^[^ ^170^ ^]^ As a connected device, the smartphone‐based thermal sensor with high resolution, sensitivity, and low cost holds great potential for in‐field applications. So far, however, few smartphone‐based thermal sensors have been used for virus detection. As the POCT market is growing and the range of applications is expanding, we believe that those acoustic, thermal or other new types of sensors will occupy an active place in the future development of smartphone‐based POC virus diagnosis.

## 4 Challenges and Future Perspectives

Laboratory‐based diagnostics methods are well developed, which remain as gold standard for virus detection. Commercial POCT technology is flourishing and is expected to be a promising alternative for virus detection. For smartphone‐based POC virus diagnostics, although many developed devices have been reported, to our knowledge, these devices or systems have not been commercialized or used in clinical setting. To investigate this issue, we summarize the current laboratory‐based diagnostics, commercial POCT, and smartphone‐based POCT for viral diagnostics in **Table** **3**. With excellent sensitivity and specificity, NAATs are still one of the most widespread diagnostic methods. Compared to NAATs, immunoassays are susceptible to antibody affinity and generally have lower sensitivity and specificity. Even so, serological tests are important because they can obtain infection history of patients who have been infected and have recovered. Until now, laboratory‐based NAATs or immunoassays remain the gold standard for virus detection. However, these assays are limited in terms of turnaround time, the need of experienced professionals, complex equipment, and the high cost of testing available to patients. Automated commercial POCT platforms transform all aspects of this workflow and minimize labor in high‐volume facilities. At the same time, commercial POCT platforms not only offer comparable sensitivity, accuracy, and specificity to laboratory‐based diagnostics, but also provide high‐throughput and flexibility by allowing multiple diagnostics and accepting more samples. Unfortunately, these commercial POCT platforms still require expensive equipment and test kits, resulting in slow market growth, especially in resource‐limited region, community, and home settings.

**Table 3:** Comparison of the presented methods for virus diagnosis

Platform	Analytical sensitivity	Clinical sensitivity	Specificity	Cost	Setting	Detecting time	Advantage	Disadvantage
RT‐PCR	1–10 copies µL^−1^	High	Moderate	10–60 USD	Laboratory	1–3 h	Highly specific, sensitive, and reliable	Sampling errors (false negatives or false positives)
RT‐LAMP	Lower than RT‐PCR	Moderate	High	Low	Laboratory	30–60 min	Low‐cost equipment without thermal alternations required	Difficult in primer design; high false‐positive results; unable of quantitative analysis
CRISPR/Cas12a/Cas 13	10–100 copies	Moderate	High	3–10 USD	Laboratory	15–30 min	No need for expertise and infrastructure; suitable for field testing	Unsatisfactory sensitivity and need for pre‐amplification
ELISA	0.01–0.1 ng	High	High	10–20 USD per test	Laboratory	2–3 h	Highly sensitive and selective with high throughput	Tedious process and sample preparation
Immunoassay	pg. mL^−1^–ng mL^−1^	High	Moderate	Low	Laboratory	10–30 min	Fast response, low cost, good flexibility, and high sensitively	Susceptible to cross‐reactive; weak stability
Miniaturized PCR device	1–10 copies µL^−1^	≥96%	≥97%	20–50 USD	Commercial POCT	1–3 h	Accurate quantification, high sensitivity and throughput	High reagent cost; expensive and complex
LOAD device	High	N/A	High	Moderate	Commercial POCT	70–150 min	Large‐scale parallelization, simple and independent liquid handling	Difficult in on‐board reagent storage, short shelf life
Lateral flow device	pg mL^−1^–ng mL^−1^	≥92.2%	≥ 95%	5–10 USD	Commercial POCT	10–20 min	Rapid; easy fabrication and transport; user‐friendly	Difficult in quantitative detection; limited capability of multiple detection
Smartphone‐based NAATs	1–100 copies µL^−1^	N/A	Moderate	Low	Field	30–80 min	Fast, simple, and high acceptable to users	Low accuracy; complex pre‐preparation process
Smartphone‐based LOC devices	Moderate	N/A	High	Low	Field	15–60 min	Easy to integration; automated result analysis; sample‐to answer detection	Difficult in fabricating, packaging, and interfacing
Smartphone‐based LFS reader	pg mL^−1^–ng mL^−1^	N/A	Moderate	Very low	Field	10–25 min	Low cost; independent on equipment; suitable for home and community testing	Highly dependent on antibody affinity and specific molecular assay; high false negatives or false positives

In response, smartphone‐based POC virus diagnostics offer a quick, simple, and cost‐effective solution for achieving broad public access to testing. As an increasingly accessible electronic device in human daily life, smartphones have opened up possibilities of POC virus diagnostics in resource‐limited areas or personalized medical management at home. The essential reason for this is the versatility, high integration, and scalability of the smartphone, which allows it to interface with a wide variety of POCT devices and replace the complex, bulky, and expensive components. For example, microfluidic POCT systems can process small volumes of parallel samples simultaneously, enabling the simultaneous, highly sensitive, and selective detection of multiple viruses. However, conventional microfluidic POCT systems require complex and expensive equipment to add samples, acquire, and analyze data. Smartphones have made it possible for microfluidic POCT systems to run sample preprocessing, molecular detection steps, analysis, and data interpretation autonomously in a sample‐to‐answer manner. Another example is the smartphone‐based LOAD system which opens up a new possibility due to its unprecedented performance in terms of time, accuracy, and cost. In the LOAD system, the liquid sample can be easily obtained by centrifugal force through a rotating motor, reducing the dependence on instruments. Smartphones can further reduce the dependence on laboratory resources and be applied to large‐scale multiplexing and parallelization at low cost, ultimately leading to a true field platform. Smartphone‐based LFA reader is also a good representative. Its low cost, ease of manufacture and operation enable rapid field testing with high reliability and low error. However, these LFS systems are still limited by the inherent shortcomings of LFS, such as low sensitivity, interference from sample matrices, high false positive/negative rates, poor multiplexing capabilities and low throughput. To address this aspect, recent smartphone‐based mobile dPCR devices offer another possibility to supply accurate absolute quantification, small sample size, and high sensitivity. When an individual's viral load is low, these devices retain their high sensitivity and accuracy while eliminating the need for large and expensive equipment components. Despite the advantages, smartphone‐based POC devices are still suboptimal in terms of sensitivity and reproducibility and remain only proof‐of‐concept in the laboratory, leaving significant challenges for achieving clinical applications.

Therefore, comprehensive improvement of assay precision and accuracy at all stages of sample preparation, signal transduction and analysis is the main focus of smartphone‐based POC virus diagnostics in the future. Improving the sensitivity and specificity of smartphone‐based POC virus diagnosis is the first challenge to provide testing results comparable to laboratory assays. Specificity improvement reduces some of the possible causes of false positives, such as the most typical nonspecific binding. Unlike high specificity, high sensitivity allows for high precision detection in very low viral load analysis, reducing false negatives as well. Continued development of better primer/probe sequences and antibodies is also an important strategy for rapid and specific identification of viral markers. Also, improving the signal‐to‐noise ratio (SNR) of the system is an important strategy to improve the sensitivity of smartphone‐based POCT systems. Optimizing the system by reducing noise or amplifying the signal allows quantification of lower concentration targets, thus improving sensitivity. In addition, signal amplification can be achieved by novel molecular diagnostic techniques (CRISPR, dPCR, dLAMP, etc.), and novel optical or electrochemical materials, such as metal nanoparticles, aggregation‐induced emission (AIE), chemiluminescent materials, molecular beacons, carbon‐based materials, and magnetic nanoparticles. Signal readout hardware for smartphone‐based POCT devices, such as CMOS cameras, often have poor performance compared to laboratory‐based hardware. Therefore, it is important to reduce noise or background signals by designing new sensing strategies or improving the performance of optical components and electrical signal processing circuits.

The second obstacle to the development of smartphone‐based POC virus diagnostics is the reliability of qualitative or quantitative detection. Smartphone‐based POCT systems often require control of the internal environment to ensure stability and reproducibility for field use. The development of calibration‐free sensors will help eliminate variations in biosensing. Improving the throughput of POCT systems can reduce clinical workload and enable testing of multiple viruses or viral samples in a single run. Therefore, full integration and development of high‐throughput smartphone‐based POCT systems with good reliability is the third challenge. Fourth, the need for standardization of smartphone‐based POC virus diagnostics is urgent in clinical practice. Smartphone‐based POC virus diagnostics should be standardized globally to collect comparable clinical results and to enable uniform analysis of test results for various diagnostic targets in different regions. We believe that any efforts to improve LOD, throughput, and diagnostic accuracy will help overcome these barriers and advance smartphone‐based POC virus diagnostics into clinical practice and commercialization.

The development of smartphone‐based POC diagnostics should begin with needs and value assessment. Researchers and clinicians could collaborate to solve an urgent diagnostic problem in a particular infectious virus disease. It must be assessed whether their devices are more valuable than currently available diagnostics techniques in terms of facilitating clinical decision‐making. To this end, the attribute value judgments for their devices should be based on three aspects: 1) the relevant molecular assay; 2) the impact of smartphone technology; and 3) cost‐effectiveness in terms of consumer demand. The effectiveness of molecular assays should be determined by the specific biomarker diagnostic requirements and validated against the current gold standard diagnostic approaches. The integration of molecular assays and mobile technology should not only increase the clinical impact of molecular assays over their laboratory counterparts, but should also keep low cost. For example, when combined with RT‐PCR, LAMP, and CRISPR technology, POC devices can improve access and reduce the time and resource burden on individuals with acute and chronic viral infections. Notably, the development of smartphone‐based POC diagnostics requires an understanding of the surrounding technical ecosystem to ensure that they integrate seamlessly into their associated socioeconomic environment.

The current widespread global COVID‐19 has once again brought attention to POC diagnostics for timely response to emerging human acute respiratory virus (RV) infections. The major RV types include influenza virus (IV), coronavirus (CoV), respiratory syncytial virus (RSV), parainfluenza virus (PIV), adenovirus, rhinovirus, and other secondary viruses that share similar symptoms, that is, cough, fever, chest pain, sneezing, and breathing difficulties^[^ ^171^ ^]^ Therefore, the clinical differential diagnosis of RV has been a biological and technical challenge. To this end, smartphone‐based diagnostics for RVs will contribute to the improvement of human health, including 1) timely tracing and isolating the infected individuals to personalized treatment. 2) accurate diagnosis of cross‐infection and reduced complication rate. 3) reducing the overuse of antibiotics (due to a lack of proper differential diagnosis of bacterial infections versus viral infections). According to the forecast by Meticulous Research, the influenza diagnostics market will reach $2 billion by 2031 (https://www.biospace.com/article/demand‐forrapid‐influenza‐diagnostic‐tests‐to‐total‐us‐2‐bn‐by‐2031/). Moreover, the rapidly growing market for RV POCTs, a shift from acute virus to chronic virus POCT strategies (e.g., HCV, HBV, and HIV) would emerge in smartphone‐based POCT technology.

Once the value of diagnostics has been fully assessed, researchers can initiate formal technology development for their devices. During this phase, technology development must ensure that it is compatible with the intended target and adheres to the WHO's REASSURED framework (real‐time connectivity, affordability, sensitivity, specificity, user‐friendliness, rapid and robust, equipment‐free, and deliverable to end‐users). An ideal smartphone‐based POC diagnostics platform should be self‐contained and automate testing and result reading. Indeed, the majority of current POC diagnostics techniques continue to rely on constant electricity, costly laboratory resources, and trained users to interpret readouts. Additionally, the hardware and software of the device should be optimized for a single version of the smartphone. The development of standalone devices with defined diagnostic components must account for a wide range of smartphone models while maintaining a tight control over the software environment. The variation in hardware and physical form factors between different brands of smartphones presents challenges for commercialization and risk assessment during the regulatory review process. Additional challenges arise from the automation and intelligence of devices. Automated result analysis provides the potential to reduce systematic and human errors associated with the interpretation, recording, and uploading of virus diagnostic test results. To accomplish this, computational and statistical methods, such as machine learning, deep learning, the Internet of Things (IoT), and other AI algorithms, are gradually integrated into custom‐built apps. Through the use of AI‐assisted systems, smartphone‐based POC diagnostics will accelerate the development of commercial POC devices, allowing for improved diagnosis and prediction of virus disease onset and progression.

Clinical validation is essential for the development of smartphone‐based POC diagnostics, which pushes this technology toward successful field trials. In this stage, the accuracy and stability of the device are two important clinical parameters to assess the clinical utility of the test. Clinical samples can vary greatly due to the patient's disease state and the patient's stage of viral infection. As a result, test results may exhibit very different performance in the device compared to the sample used for proof of concept. These POC devices need to be validated in clinical diagnostics to meet their intended end use. Finally, technological innovations in mobile POC diagnostics must comply with regulatory policies, such as the Food and Drug Administration (FDA), the European Commission and the WHO. These guidelines ensure the quality of the commercialization process of smartphone‐based POC diagnostics, including technology development, device manufacturing, end‐use, and consumer services.

## 5 Conclusions

The convergence of mobile technology and the field POCT diagnostics offers potential opportunities for the development of groundbreaking technologies for infectious virus diagnosis. Advanced strategies of smartphone‐based POC diagnostics have enabled laboratory‐based molecular techniques such as immunoassay and nucleic acid amplification to be performed in plug‐and‐play stand‐alone devices. Meanwhile, smartphone technology is disrupting the traditional field of infectious virus diagnostics by democratizing and decentralizing testing, as well as increasing their accessibility in resource‐limited settings. Automated and intelligent mobile‐assisted testing with equipment‐free diagnostic devices is rapidly penetrating into private homes and personalized testing due to the potential of public participation. Additionally, the real‐time connectivity of smartphones will facilitate the development of next‐generation POC technology, allowing individuals to access digital health pathways for integrated virus outbreak detection, surveillance, and disease epidemic containment. However, only a few proof‐of‐concept POCT devices have been fully integrated and deployed, and their efficacy has not been evaluated on a large scale. To accomplish this, researchers must assess the needs and value, the cost, the development of technology, the clinical effectiveness, and the applicable regulation. Otherwise, smartphone‐based POC technologies will face unpredictable barriers to commercialization, institutional adoption, and public confidence. The future of smartphone‐based POC diagnostics is bright, and it is changing the way patients are diagnosed in centralized laboratories. At the same time, advances in smartphone‐based POC technology could set the stage for a larger market in global public health. We believe this review can help push the development of mobile diagnostics forward with scientific and available guidance, leading to more valuable devices and ultimately helping to reduce the global burden of infectious disease.

## Conflict of Interest

The authors declare no conflict of interest.

## Author Contributions

M.X., F.T., and X.L. contributed equally to this work. M.X. is responsible for the manuscript writing of Sections 1 and 3. X.L. is responsible for the manuscript writing of Section 2. F.T. is responsible for the manuscript writing of Sections 4 and 5. Q.Z. and J.P. assist in literature collection and manuscript writing. C.Y. is responsible for the design of manuscript structure. Z.L, M.Y, and C.Y. are responsible for guiding, reviewing, and revising the manuscript. All authors agree with this arrangement of the author sequence in this, manuscript.
